# Regular Tennis Exercise May Improve the Vascular Endothelial Function in Postmenopausal Women: The Influence of Hemodynamics

**DOI:** 10.3390/ijerph192315749

**Published:** 2022-11-26

**Authors:** Weifeng Pan, Lijing Gong, Guoan Xiao, Lantian Zhang, Yiran Xiao, Chunyan Xu

**Affiliations:** 1 Key Laboratory of Exercise and Physical Fitness, Ministry of Education, Beijing 100084, China; 2 China Institute of Sport and Health Science, Beijing Sport University, Beijing 100084, China; 3 Beijing No.10 Middle School, Beijing 100039, China; 4 Sport Science College, Beijing Sport University, Beijing 100084, China

**Keywords:** exercise prescription, atherosclerosis prevention, tennis exercise, endothelial function

## Abstract

Physical inactivity plays a role in the incidence of cardiovascular disease (CVD). Although the current guidelines for physical activity, such as the prescription of exercise, seek to combat CVD, attaining the recommended targets remains challenging. Tennis exercise has been proven to have a unique advantage in reducing the mortality of CVD, but little is known about the influence of playing tennis on impaired vascular endothelial function (VEF), which initiates CVD. Thus, this study aimed to investigate whether regular tennis participation could protect the VEF better than merely meeting the physical activity recommended by the current guidelines. A cross-sectional design was performed based on a sample of 38 healthy postmenopausal women who were matched for physical activity, of which 17 subjects had long-term tennis experience and 21 age-matched subjects regularly exercised but did not play tennis. The cardiovascular function and the body composition of all subjects were measured. We used cluster analysis to assess the overall health status. The modeling results showed that the tennis players performed better in terms of VEF than the nonplayers (10.55 ± 0.58 vs. 8.69 ± 0.52, *p* < 0.01, *R^2^_ad_* = 0.367), while the wall shear stress positively correlated with VEF (r = 0.505, *p* < 0.05), after controlling for age and physical activity levels. Regular tennis exercise may be a protective factor for VEF, and further study should be performed to research the role of hemodynamics in tennis exercise.

## 1. Introduction

Cardiovascular disease (CVD) remains the major cause of death worldwide [1], and risk factors such as physical inactivity (PI) combined with sedentary behavior (SB) play a role in CVD mortality [2,3,4]. Overwhelming evidence suggests that the greater the physical activity (PA), the lower the all-cause mortality and CVD mortality, and cardiometabolic risk factors show a decreasing tendency with increasing physical activity [5,6]. Although overwhelming evidence exists demonstrating that following a physically active lifestyle is associated with promoting health, many efforts to comply with this advice fail, and even the minimum 150 min of physical activity per week recommended by the World Health Organization cannot be reached; about one third of adults worldwide are classified as sedentary or physically inactive; in particular, this percentage is apparently higher in developed countries, and aging countries regardless of region [7]. Since physically active individuals normally display better body composition, musculoskeletal function, and cardiorespiratory fitness, the overall enhanced functionality evidently reduces chronic diseases, such as atherosclerosis, across the life span [4]. Hence, more feasible methods to reach the target physical activity are needed.

Exercise prescription is naturally considered as a solution to combat the ever-growing rates of the emerging CVD risk factors; however, this is often associated with little knowledge about the underlying mechanisms and plausible recommendations, resulting in futile efforts [8]. This emphasizes the need for more feasible and sustainable exercise prescription which can directly promote specific cardiovascular health across the life span. Currently, most studies on exercise prescription concentrate on aerobic, strength, and combined training, while optimal exercise prescription should integrate strength, stability, flexibility, aerobic, and anaerobic training [9]. A previous study has shown the extraordinary effectiveness of group-based exercise prescription for adults, which leads to higher program compliance and exercise adherence [10].

Oxford University recently conducted a prospective cohort study of 80,306 UK adults, investigating the association of specific types of exercise with all-cause and CVD mortality, and found that racquet sports could reduce all-cause mortality by 47% and CVD mortality by 56%, which ranked first among the included exercises in the study. Another longitudinal study revealed that tennis exercise could significantly reduce the CVD risk factors compared with other racquet sports [11]. A recent review indicates that compared with age-matched individuals, tennis players displayed significantly higher fitness levels, leaner bodies, and better cardiovascular health [12]. Tennis is a sport that integrates various modes of aerobic and sprint exercise due to the nature of the competition and the combination of the necessary skills, all of which are in a group-based environment [13], resulting in a series of physiological stimulations during the game [14]. Nonetheless, there remains a lack of study investigating the effects of regular tennis exercise on CVD specifically, despite the many health benefits already reported [15,16]. Many studies have not considered the difference in the physical activity levels; hence, the health benefits could not be independently attributed to tennis exercise. Moreover, Oja et al. controlled the confounders such as physical activity levels in their reviews, but they did not research the difference among racquet sports or the mechanism of tennis exercise on reducing CVD mortality [11]. However, it is evident that understanding the physiological response to specific exercises can help us to prescribe exercise efficiently [8,9].

Atherosclerosis, as a major cardiovascular disease, is associated with a high number of deaths worldwide, rather than being concentrated in Western countries as before. Notably, its risk factor characteristics have changed in recent years, in which the traditional risk factors have been reduced, meanwhile, new risk factors, such as PI and SB, are receiving increasing attention [17]. Previous studies have suggested that decreased vascular endothelial dysfunction initiates cardiovascular and cerebrovascular diseases and is one of the earliest signs of arteriosclerosis, which has been proven to be an independent predictor of cardiovascular disease [18,19]. Additionally, there is sufficient evidence that vascular endothelial dysfunction is convertible; regular exercise can remodel the vascular structure while varying the exercise types can lead to different adaptions [20,21,22,23].

Endothelial function is associated with menopause. The risk of CVD is significantly increased due to losing the protection of estrogen in postmenopausal females [24,25]. A recent study suggested that the menopausal transition is associated with an increase in fat mass (predominantly in the truncal region), along with an increase in insulin resistance, dyslipidemia, and endothelial dysfunction [26]. Thus, there exists a practical need to investigate the protective effect of tennis exercise on the endothelial function in postmenopausal women. We hypothesized that tennis players would display a significantly healthier endothelial function than physically active nonplayers.

## 2. Materials and Methods

### 2.1. Participants

The study protocol was designed in accordance with the recommended principles for ethics set out by the Declaration of Helsinki, and ethical approval was granted by the institutional ethics committee. Informed written consent was obtained from each of the participants. The G*Power [27] software was used to determine the sample size with 0.8 power and 0.05 α error probability (two tailed), based on previous research, which elaborated a significantly higher flow-mediated dilation in tennis players versus weightlifters [28]. Therefore, a total sample size ≥34 participants was required to observe a faithful significant difference. To ensure this was obtained, a slightly higher number of participants were recruited; finally, 38 female participants, aged from 50 to 71 years, took part in the research. All the participants were recruited from Beijing, near to the Beijing Sport University. The tennis players (n = 17) had an average of 13.46 ± 6.52 years of playing experience and took part in tennis at least two times per week. All the tennis players who were willing to enroll in this research were amateur tennis players, none were elite. A group of age-matched nonplayers were recruited from the same region. All the participants were in normal health with no health-related complaints or cardiometabolic disorders present. Although from a variety of exercise backgrounds, all nonplayers who regularly exercised met the recommended physical activity by the World Health Organization (150 min of moderate to vigorous physical activity per week [4]), demonstrated by their responses to the International Physical Activity Questionnaire. Therefore, the physical activity between groups was matched.

### 2.2. Study Design

An information sheet with the entire procedure was provided to each participant, and once agreed, all offered their written informed consent. Participants were later required to accomplish a physical activity willingness questionnaire to confirm no contraindication to sport. All procedures were accomplished in the visit to the China Institute of Sport and Health Science of Beijing Sport University and were performed by the same examiner. Participants were required to abstain from any food in the 2–4 h prior and to not consume any caffeine, alcohol, or drugs or participate in any vigorous physical expenditure in the 24 h prior to testing.

### 2.3. Anthropometry

Height and weight were measured by an electric stadiometer, and all participants were instructed to wear only light clothes and stand straight with bare feet. The body mass index (BMI) was calculated via the formula BMI = Weight (KG)/Height(m). Body composition was measured by Dual X-ray absorptiometry (DXA, XR-46, Norland, USA), and the apparatus was calibrated prior to the test. The basic information of the participants was entered into the device, and participants were instructed to wear only light clothes, take off any iron items, and lie down the on the center of the scan stage in a supine position. The lower extremities were internally rotated together, while the toes were placed side by side, and the arms were placed close to the body with the palms down; then, both upper extremities and lower extremities were fixed by tape. The signals were then calculated by the computer, which exported the parameters of the body fat percentage (BFP), body fat mass (BFM), lean body mass (LBM), and bone mineral density (BMD). By using the DXA, two different energy rays could be obtained at the same time, which could clearly display the body fat distribution of whole body and accurately measure the parameters of body composition.

### 2.4. Cardiovascular

The double side of the carotid intima-media thickness (CIMT) and the cardiac parameters were measured via color Doppler echocardiography(LOGIQe, GE, Connecticut, USA). When testing the CIMT, participants were instructed to urinate and then rest for 5 min prior; then, they laid down in the supine position, slightly lifted the chin, and turned the head to the non-tested side to allow the carotid to be explored adequately. Then, the 12L-tiyu-type probe used to perform the test was coated with couplant and placed on the carotid; then, we found the longitudinal of the vessel clockwise and scanned along the outer edge of sternocleidomastoid muscle. The test was repeatedly performed three times for each side, and the highest value on each side was chosen to represent the carotid intima-media thickness. To assess the cardiac function, participants were then instructed to lie on their side, and the probe was placed on the left edge of the 3–4 sternum pointing to the shoulder, and we entered the “probe” key to switch to the M4-type probe. The probe was first placed between the shoulders and waist and then moved around the cardiac area to locate the apical, for the purpose of scanning the cardiac area accurately to obtain a clear image. The stroke volume (SV), cardiac output (CO), left ventricular end diastolic volume (LVEDV), left ventricular end stroke volume (LVESV), ejection fraction (EF), peak systolic velocity (PSV), and carotid artery end diastolic diameter (CAED) were exported. The stroke volume index (SVI), cardiac index, and wall shear stress (WSS) were then calculated via the following formulas: 


$$SVI=SV\left(\text{mL}\right)÷BSA\left({\text{m}}^{2}\right)$$

$$CI=CO\left(\text{L}/\text{min}\right)\;÷\;BSA\left({\text{m}}^{2}\right)$$

$$\text{body}\;\text{surface}\;\text{area}\;(\text{BSA})=0.00844673\times {\text{Weight}}^{0.4167}\times {\text{Height}}^{0.6997}$$

$$\text{WSS}=4\times \eta \;\times \frac{\mathrm{PSV}}{\mathrm{CAED}}$$

BSA (body surface area) [29].

WSS (wall shear stress) [30].

η (blood viscosity) = 4.

### 2.5. Vascular Endothelial Function

Vascular endothelial function was measured via an UNEX EF apparatus(38G, Omron, Osaka, Japan), adopting the standard guidelines for ultrasound assessment of endothelial-dependent flow-mediated vasodilation (FMD) of the brachial artery [31]. Since the FMD is affected by confounders such as temperature, food, drugs, and sympathetic stimulation, participants were required to abstain from caffeine, alcohol, a high fat diet, nicotine, and vitamin C and to not have exercised vigorously in the 24 h prior. On the testing day, the apparatus was calibrated beforehand, and participants were instructed to lie down on the testing stage in a supine position in a cool and quiet room. After 10 min rest, participants were instructed to put their arms near to the body, whilst the blue compression band was tied to the position above the elbow joint, and two clips were clamped on the wrist of the subject’s left arm. The right arm was freely placed on the mat of the operating desk, and the red compression band was tied to the position above the wrist, with the palm fixed on the operating desk. The tightness between the band and arm through which two fingers could be fitted was considered eligible. Couplant was coated on the humerus about 3–5 cm above the elbow joint of subject’s right arm, and the Type-H probe was then located on the brachial artery. When a clear image emerged on the screen, the probe was fixed, and the resting brachial vessel diameters with blood flow velocity were measured. At the end of the measurement, the subject was required to remain motionless, given a 50 mmHg boost based on quiet blood pressure, and performed 5 min of exercise. After 5 min, the pressure was automatically released, whilst the inner diameter and blood flow velocity within 2 min were automatically recorded, and the FMD was calculated by computer.

### 2.6. Vascular Stiffness 

An Arteriosclerosis detector (BP−203RPEI, Omron, Osaka, Japan) was used to assess the vascular stiffness. The temperature of the room was controlled at 22–25 °C, and subjects were required to urinate in advance, and to abstain from nicotine, drugs, caffeine, and food. Then, the subjects were instructed to wear light clothes, and the ankle was exposed, while the subject laid in a supine position. The examiner explained to subjects the measurement method to relieve the subjects’ nervousness and instructed the subjects not to speak or move their body during the test. After resting for 5 minutes, measurement bands were tied to the upper arm and the position above the ankle joint, respectively. The electrodes of the ECG and heart sound sensors were mounted, and the ECG sensor was fixed above the wrist while the heart sound sensor was placed in the center of the fourth rib on the left side of the chest, and the heart sound intensifier was placed on top. The measurements were repeated two times and were stopped immediately if the subject could not tolerate pressurization during the measurement. The brachial-ankle pulse wave velocity (baPWV) and ankle-brachial index (ABI) were exported.

### 2.7. Statistical Analysis

The data are presented as means ± standard deviations or means ± standard errors as appropriate. First, abnormal data were identified via the Grubbs criterion, Dixon criterion, and Romanovsky test, from which significant abnormal values were excluded. All the variables were second tested for normal distribution and equal variance, following comparison between the tennis players’ group and the nonplayers’ group using an independent-samples t-test, which was performed where the data were normally distributed and had equal variance. Moreover, in some cases, where a normal distribution or equal variance was not available, a nonparametric analysis Mann–Whitney U test was performed instead of the t-test. To analyze the potential correlation between variables and age, the Pearson correlation was used where data were normally distributed. Additionally, the Spearman correlation was performed where data were nonnormally distributed. Furthermore, in order to investigate the probability mechanism that tennis exercise reduced the risk of cardiovascular disease, partial correlation analysis between the variables and FMD was performed, using age as a control variable.

Overall cardiovascular and body composition function was then analyzed via cluster-based techniques following the previous method set by Matthew and Andersen et al. [32,33]. The variables were first classified via R-variance clustering, and three clusters were generated to assess the cardiac, vascular, and body composition function, respectively. Cluster 1 (C1), the relative basic cardiac health component, was represented by the HR, SVI, CI, SV, CO, and EF; Cluster 2 (C2), the relative vascular health component, was represented by the FMD, WSS, CIMT, PSV, CAED, ABI, and baPWV; Cluster 3 (C3), the relative body composition, was represented by the BMI, BFP, BFM, and LBM.

All selected variables were then converted into z-scores, and to ensure each component had the same directional weighting, the HR was multiplied by −1 to switch the z-scores into a positive weighting in C1; the CIMT, CAED, and baPWV were multiplied by −1 to switch the z-scores into a positive weighting in C2; and the BMI, BFM, and BFP were multiplied by −1 to switch the z-scores into a positive weighting in C3. The cluster scores of the cardiac health component, vascular health component, and body composition were then calculated by the summation of their respective factors, and the greater the cluster score, the greater the cardiac or vascular health. For the comparison between the tennis players and nonplayers, analysis of covariance (ANCOVA) was performed, using age as a covariate. *p* < 0.05 was considered as statistically significant. SPSS Statistics software (version 25.0, Chicago, IL, USA) was used to analyze all data.

## 3. Results

**Figure 1:**
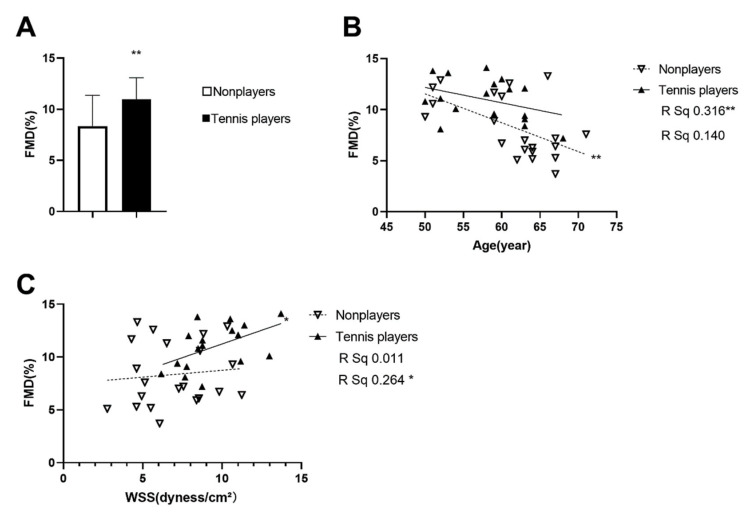
* p < 0.05 and ** *p* < 0.01 were considered statistically significant. Comparison of the flow-mediated dilation (FMD) between the tennis players and nonplayers (**A**); correlation of the FMD with increasing age between the tennis players and nonplayers (**B**); correlation of the FMD with increasing vessel wall shear stress (WSS) between the tennis players and nonplayers (**C**).

Table 1 displays the descriptive characteristics of the tennis players and nonplayers. The tennis players showed a significantly lower BMI, BFP, BFM, and higher LBM than nonplayers in the anthropometry parameters (*p* < 0.05); with respect to the cardiac parameters, the tennis players displayed a significantly lower HR than nonplayers (*p* < 0.05); as for the hemodynamic parameters, a higher PSV and WSS were observed in the tennis players (*p* < 0.05); regarding artery stiffness parameters, the tennis players had a significantly lower FMD and left-CIMT than the nonplayers (*p* <0.05). Figure 1A shows the difference in the FMD between the tennis players and nonplayers.

As shown in Table 2, the simple correlation analysis revealed that the BMI and BFM showed a significantly positive correlation with age, whilst the FMD and WSS displayed a significantly negative correlation with age in the nonplayers (*p* < 0.05). Notably, the BMI, BFM, and FMD showed varying degrees of correlation with age in the nonplayers (*p* < 0.01). No significant correlations with age were observed in the tennis players. Table 3 and Table 4 indicated the correlation between the body composition, cardiovascular parameters, and FMD after controlling for age. Figure 1B highlights the comparison of FMD with increasing age, which indicated the tendency of the FMD to decrease with increasing age.

**Figure 2:**
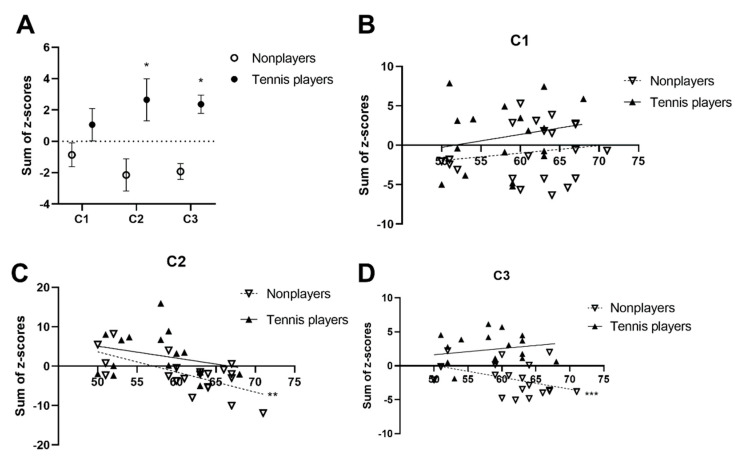
* p < 0.05 was considered statistically significant. ** *p* < 0.01 and *** *p* < 0.001 indicate a varying level of significance. Comparison of the cluster scores between the tennis players and nonplayers (**A**); Correlation of C1, C2, and C3 with increasing age between the tennis players and the nonplayers (**B**–**D**). Cluster 1 (C1) represents the cardiac function and is the summary of the z-scores for the HR, SVI, CI, SV, CO, and EF; Cluster 2 (C2) represents the arterial function and is the summary of the z-scores for the FMD, WSS, CIMT, baPWV, ABI, PSV, and CAED; Cluster 3 (C3) represents the body composition function and is the summary of the z-scores for the BMI, BMP, BFM, and LBM.

Without considering age, the correlations among the variables revealed that the FMD positively correlated with wall shear, while it negatively correlated with the left baPWV and CAED in the tennis players (*p* < 0.05), but no significant correlations with the FMD were observed in the nonplayers. Partial correlation analysis, adjusted by age, then revealed that the FMD still positively correlated with the WSS and negatively correlated with the CAED, left CIMT, and left baPWV in the tennis players (*p* < 0.05). Once again, no significant correlations between the variables and the FMD were observed in the nonplayers. Figure 1C displays the correlation of the FMD and WSS adopting linear correlation analysis between the different groups. Table 5 displays the results of ANCOVA on the FMD, WSS, Cluster 1, Cluster 2, and Cluster 3, using age as a covariate. For analysis of the overall cardiovascular and body composition health, three clusters were generated from the markers of cardiac, vascular, and body composition data. Specifically, all the selected variables were standardized in z-scores and then summed to provide measures of the cardiac, vascular, and body composition function. In order to assess the cardiac function, the HR was combined with the SVI, CI, SV, CO, and EF to generate C1. There was no significant difference in the cluster scores between the tennis players and nonplayers. Then, for the purpose of assessing the vascular function, the FMD was combined with the WSS, CIMT, ABI, PSV, CAED, and baPWV to generate C2. As hypothesized, the tennis players did show significantly higher z-scores than nonplayers in this aspect (nonplayers, −1.51 ± 1.01 [n = 21] versus tennis players, 1.87 ± 1.12 * [n = 17], $R{²}_{ad}$ = 0.341; *p* < 0.05). Moreover, to assess the body composition, the BMI was combined with the BFP, BFM, and LBM to generate C3. Once again, the tennis players displayed significantly higher z-scores than nonplayers (nonplayers, −1.81 ± 0.52 [n = 21] versus tennis players, 2.24 ± 0.58 *** [n = 17], $R{²}_{ad}$ = 0.45; *p* < 0.05). Figure 2A displays the comparison of C1, C2, and C3 between the tennis players and nonplayers.

Table 6 and Figure 2B−D show the correlation between age and Cluster 1, Cluster 2, and Cluster 3. There was no significant correlation between C1 and age, while C2 and C3 negatively correlated with age in the nonplayers. No significant correlations between all three clusters and age were observed in the tennis players. 

**Figure 3:**
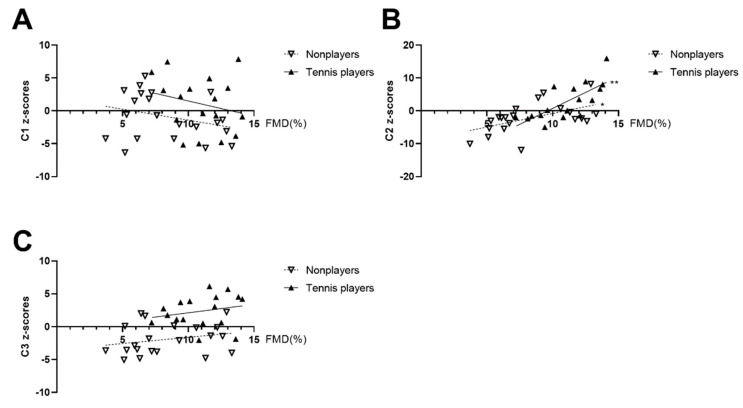
* *p* < 0.05 was considered statistically significant. ** *p* < 0.01 indicates a varying level of significance. Correlation of C1, C2, and C3 with increasing FMD between the tennis players and the nonplayers(**A**–**C**). Correlation of the cluster scores with increasing flow-mediated dilation between the tennis players and nonplayers. Cluster 1 (C1) represents the cardiac function and is the summary of the z-scores for the HR, SVI, CI, SV, CO, and EF; Cluster 2 (C2) represents the arterial function and is the summary of the z-scores for the FMD, WSS, CIMT, baPWV, ABI, PSV, and CAED; Cluster 3 (C3) represents the body composition function and is the summary of the z-scores for the BMI, BMP, BFM, and LBM.

**Figure 4:**
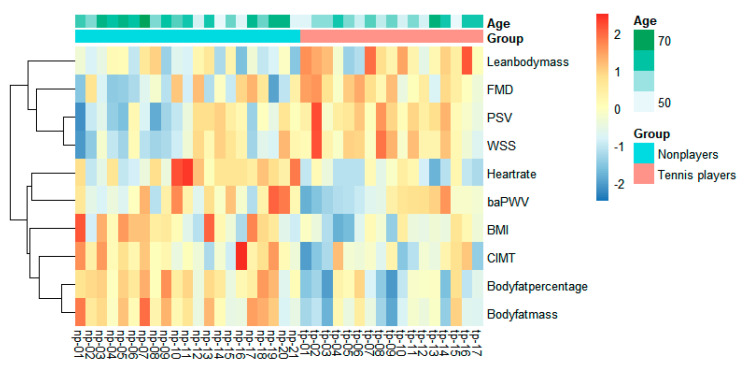
Clustered tendency of the measured parameters between the tennis players and nonplayers.

Table 7 displays the correlation between the FMD and Cluster 1, Cluster 2, and Cluster 3. Figure 3 indicates the FMD positively correlated with C2 in the tennis players, and no significant correlation was observed after controlling for age. Figure 4 displays the result of the cluster analysis using the original measured parameters, which shows the clustered tendency between tennis players and nonplayers.

## 4. Discussion

Previous studies have shown that tennis exercise promotes longevity; meanwhile, significantly lower all-cause and CVD mortality have been reported in those who have played tennis over the long term [34,35]. However, little is known about effects of tennis exercise on the vascular endothelial function (VEF). The nature of VEF regulation is to maintain the dynamic balance of vasoconstriction and dilation; therefore, maintaining normal vascular endothelial function is an important condition for vascular health [19,36]. Many studies suggest impaired VEF, proven as the start of atherosclerosis, which is the leading cause of coronary disease, can be an independent predictor for CVD [19,37]. Vascular endothelial dysfunction is mainly manifested as decreased vasodilation. Clinical studies have confirmed that ultrasound detection of the FMD can independently predict the risk of cardiovascular events [38,39]. Owing to its noninvasive and reproducible characteristics, FMD is widely used in clinical research to predict long-term cardiovascular events in subjects without heart disease [40]. 

Menopause is an important risk factor, which is evidently associated with endothelial dysfunction [41,42]. The incidence of coronary heart disease in premenopausal women is about 1/10 that of men, and in postmenopausal women, it rapidly increases to close to that of men [24]. Basic studies have elucidated that estrogen treatment prevents apoptosis and necrosis of cardiac and endothelial cells. Estrogen also ameliorates pathologic cardiac hypertrophy, which indicates that estrogen may have a great benefit in aging as an anti-inflammatory agent [25]. Evidence from a meta-analysis suggested that low-dose estrogen hormone therapy can reduce the risk of CVD in postmenopausal women via promoting a better lipid profile [43]. These findings reveal that menopausal transition is associated with an increase in fat mass (predominantly in the truncal region), an increase in insulin resistance, dyslipidemia, and endothelial dysfunction [26]. Additionally, hemodynamic factors directly act on the vessel wall, and thus their impacts on vascular function have attracted attention in recent years [44,45]. Many studies have supported the correlation between impaired VEF and abnormal low WSS [46].

Regular exercise can significantly improve the risk factors for endothelial dysfunction and even remodel the vascular structure [20,21,23]. The literature illuminates that exercise can improve vascular endothelial function in various ways, such as reducing blood lipids, blood glucose, and blood pressure levels, promoting nitric oxygen release, etc. [47,48]. Tanahash et al. [49] conducted 12 weeks of aerobic training in 102 adults and found that the brachial artery WSS increased after intervention. Green et al. [21,23] recently demonstrated that artery function changed immediately during acute exercise by the stimulation of hemodynamic factors, whereas chronic functional adaption was probably induced by repeatedly being exposed to episodic bouts of such stimuli.

Thus, it could be assumed that tennis exercise could promote a better VEF compared with other types of physical activity, and this improvement may correlate with hemodynamic factors. In the present study, the apparatus which was used to measure the FMD could correct the error that traditional manual measurement may miss, the maximum inner diameter, and it has been applied in many large sample studies, which have verified its unique advantages [50]. 

The result of the tests showed that the tennis players did display a significantly higher FMD and WSS than the physically active nonplayers; both the FMD and WSS were negatively correlated with age in the nonplayers, which was not observed in the tennis players; the FMD was positively correlated with the WSS in the tennis players, while no such correlation was found in the nonplayers. The overall vascular health represented by the C2 score was significantly higher in the tennis players than the nonplayers when compared by cluster analysis; furthermore, the C2 score was negatively correlated with age in the nonplayers, and no significant correlation was observed in nonplayers. The FMD was positively correlated with C2 in the tennis players, but no such significant correlation was observed in the nonplayers after adjusting for age. The results suggested that compared with other exercise methods, tennis exercise could better (i) promote the health of vascular endothelial function; (ii) delay vascular aging, which may be attributed to enhanced endothelial function; and (iii) improve the blood flow dynamic conditions, which may be one of the reasons for its improved endothelial function. The superior VEF observed in tennis players, to a certain extent, accounted for the unique effects of tennis exercise on reducing CVD mortality. In addition, the correlation between the FMD and WSS found in the present study further proves that hemodynamic factors play a large role in the exercise-induced change of endothelial function [21,23]. 

Although there is much convincing evidence to support the maintenance and enhancement of cardiovascular health by tennis exercise [15,51], the effects of tennis exercise on overall vascular function compared with other means of exercise have rarely been reported. In this study, when considering the FMD, CIMT, baPWV, ABI, WSS, CAED, PSV, and other indicators that could reflect the degree of vascular stiffness, the tennis players showed healthier overall vascular function, and vascular aging was delayed. However, it remains unclear why tennis exercise has such a unique effect on reducing cardiovascular risk compared with other exercise. Previous studies have shown that moderate intensity exercise can reduce CVD mortality, and high-intensity exercise is superior to moderate-intensity exercise in reducing the risk of cardiovascular disease [11]. In addition, resistance training is associated with muscle fatigue resistance and heart health benefits [52,53]. Obviously, this evidence suggests that hybrid training, defined by ACSM as a compound training that combines various training modalities, provides the best conditions for promoting healthy adaptation [9,54]. However, this does not seem to explain why, in this study, the overall vascular function of some nonplayers following this criterion was still significantly lower than that of the tennis players, evidenced by better C2 scores, which is consistent with Oxford’s study [34]. Longer duration in a tennis match probably accounts partly for this [14,55]. Most tennis matches will last more than 1 h, including short bursts of high-intensity exercise (4–10 s) and minimal rest periods (10–20 s), with typically 300 to 500 intensity workouts per match [56]. Comprehensive bursts of high-intensity exercise led to a modest physiological response, equivalent to an intensity of 60 to 80 percent of maximum heart rate, 60 to 70 percent of maximum oxygen consumption, and a build-up of 2 to 4 mmol/L of blood lactate [57,58]. Combined with minimal rest periods, the high metabolic challenge to the body is induced by the high work-to-rest ratio [14,55]. The nature of tennis exercise, combining aerobic, strength, and high-intensity intervals with repeated sprints, with a long single exercise time means that not only is the amount of the activity sufficient, but the exercise compliance rate is also high, and long-term stimulation is conducive to chronic adaptation [13,59].

Although many studies have reported the health benefits of tennis exercise, the cluster analysis was conducted to illustrate the potential of tennis exercise to enhance cardiovascular fitness for the first time. Because of the presence of a physically active control group, the benefits of these exercises can be independently attributed to tennis exercise, rather than to increases in physical activity. This study considered the effect of age, and many measures had a statistical difference when compared via cluster analysis, which might be due to the contribution of the marginal differences of each measure. Importantly, this finding specifically highlights its potential in improving vascular endothelial function, where new and more practical alternatives to existing physical activity recommendations are needed.

There were some limitations in this study. First, due to the prevalence of COVID−19, we could not recruit more participants to include a blank control group for physical inactivity. Second, because of the nature of the cross-sectional design, it can only be hypothesized that the differences between groups were caused by the different type of physical activity rather than other underlying factors. To mitigate this, we made some effort to control for confounders such as age and levels of physical activity. However, profession and income levels may also be potential confounding variables, and we could not collect information on social status in this study. Thus, we could only rely on the recent literature to prove indirectly that tennis exercise was an independent affecting factor, not related to economic conditions [60]. Third, although we found that hemodynamic factors play an important role in improving vascular endothelial function, the hemodynamic indicators in this study were relatively simple and were not sufficient to comprehensively evaluate complex hemodynamic conditions. Fourth, due to the limited conditions, the indicators included in the cluster analysis only partially reflected the cardiovascular function. Finally, although the control group in the experiment was considered to be physically active enough to meet at least the minimum recommended amount, we did not collect information on the specific type of physical activity chosen by them, nor did we track their physical activity and sedentary behavior levels over time.

## 5. Conclusions

The tennis players had a higher VEF than the nonplayers (10.55 ± 0.58 vs. 8.69 ± 0.52, *p* < 0.01, R^2^_ad_ = 0.367), while the wall shear stress positively correlated with the VEF (r = 0.505, *p* < 0.05) after controlling for age and physical activity levels. This study provides evidence for tennis as a protective factor from endothelial dysfunction in postmenopausal women, and hemodynamic factors play a role in this process. This may be attributed to the nature of the mixed and intermittent high-intensity exercise of this game. In addition, the competitive atmosphere and enjoyment during the game may partially explain the higher exercise compliance rate in tennis, which is also essential for exercise prescription. These findings indicate that tennis is an effective physical activity pattern to combat CVD via ameliorating VEF and should consequently be more recommended as a feasible alternative to current physical activity guidelines.

Longitudinal studies, such as prospective cohort studies, are warranted, which may explain many confounding variables. Future studies should try to control the income levels and monitor the physical activity of participants and information about specific types of physical activity and sedentary behavior should be collected.

## Figures and Tables

**Table 1:** Comparison of characteristics between the tennis players and the nonplayers.

	Nonplayers	Tennis Players
	n = 21	n = 17
Age, year	61.33 ± 5.98	58.06 ± 5.25
Height, m	160.95 ± 6.61	163.82 ± 5.14
Weight, kg	60.34 ± 5.83	58.13 ± 4.32
BMI, $\mathrm{kg}\cdot m^{-2}$	24.03 ± 2.61	21.88 ± 1.75 **
BFP, %	33.96 ± 4.86	24.51 ± 7.00 ***
BFM, kg	21.31 ± 4.84	14.47 ± 4.55 ***
LBM, kg	39.71 ± 3.39	44.21 ± 4.64 **
Vigorous PA, MET-min/w	441.72 ± 162.06	386.00 ± 79.76
Moderate PA, MET-min/w	1691.05 ± 821.11	1589.71 ± 265.23
Walk PA, MET-min/w	1078.48 ± 538.30	1218.70 ± 527.75
Total PA, MET-min/w	3005.20 ± 787.74	3132.01 ± 425.66
SV, mL	57.94 ± 23.07	64.59 ± 27.62
CO, L/min	3.53 ± 1.47	3.80 ± 1.57
EF, %	72.64 ± 13.13	71.23 ± 8.01
SVI, $\mathrm{mL}\cdot m^{-1}$	35.03 ± 14.15	39.62 ± 17.25
CI, L/(min/m²)	2.13 ± 0.88	2.33 ± 0.98
CAD, mm	6.07 ± 0.74	5.86 ± 0.60
PSV, $\mathrm{cm}\cdot s^{-1}$	25.94 ± 7.25	34.13 ± 5.29 ***
WSS,$\;\mathrm{dynes}\cdot {\mathrm{cm}}^{-2}$	6.95 ± 2.41	9.49 ± 2.09 **
FMD, %	8.35 ± 3.01	10.97 ± 2.12 **
Left CIMT, mm	0.73 ± 0.10	0.64 ± 0.10 *
Right CIMT, mm	0.66 ± 0.14	0.62 ± 0.09
Left baPWV, $\mathrm{cm}\cdot s^{-1}$	1452.25 ± 164.04	1422.29 ± 212.46
Right baPWV, $\mathrm{cm}\cdot s^{-1}$	1486.43 ± 189.07	1411.67 ± 218.86
Left ABI	1.24 ± 0.09	1.22 ± 0.06
Right ABI	1.23 ± 0.09	1.24 ± 0.09

The data are presented as mean ± SD, and * *p* < 0.05, ** *p* < 0.01, *** *p* < 0.001 were considered statistically significant. BMI, body mass index; BFP, body fat percentage; BFM, body fat mass; LBM, lean body fat; PA, physical activity; HR, heart rate; LVEDV, left ventricular end diastolic volume; LVESV, left ventricular end systolic volume; SV, stroke volume; CO, cardiac output; EF, ejection fraction; SVI, stroke volume index; CI, cardiac index; FMD, flow-mediated dilatation; CAD, carotid artery diameter; PSV, peak systolic velocity; WSS, wall shear stress; left CIMT, left carotid artery intima-media thickness; right CIMT, right carotid artery intima-media thickness; right baPWV, brachial-ankle pulse wave velocity; left baPWV, left brachial-ankle pulse wave velocity; right ABI, right ankle brachial pressure index; left ABI, left ankle brachial pressure index.

**Table 2:** Correlations between the measured parameters and age between the different exercise groups.

	Nonplayers	Tennis Players
BMI	0.591 **	−0.336
BFM	0.568 **	−0.132
FMD	−0.563 **	−0.374
WSS	−0.434 *	−0.132

* *p* < 0.05, ** *p* < 0.01 were considered statistically significant. BMI, body mass index; BFP, body fat percentage; BFM, body fat mass; LBM, lean body fat; FMD, flow-mediated dilatation; WSS, wall shear stress.

**Table 3:** Correlations between the FMD and the body composition parameters.

	Simple Correlation Analysis		Partial Correlation Analysis Adjusted by Age	
	Nonplayers	Tennis players	Nonplayers	Tennis players
BMI	−0.406	−0.224	−0.109	−0.401 *
BFP	−0.200	−0.218	0.017	−0.382 *
BFM	−0.224	−0.265	0.141	−0.442 *

* *p* < 0.05 was considered statistically significant. BMI, body mass index; BFP, body fat percentage; BFM, body fat mass.

**Table 4:** Correlations between the FMD and the cardiovascular parameters.

	Simple Correlation Analysis		Partial Correlation Analysis Adjusted by Age	
	Nonplayers	Tennis players	Nonplayers	Tennis players
CAED	−0.039	−0.586 *	0.187	−0.544 *
WSS	0.104	0.513 *	−0.189	0.505 *
Right CIMT	−0.021	−0.456	0.282	−0.589 *
Left baPWV	−0.311	−0.567 *	−0.245	−0.507 *
Right baPWV	−0.354	−0.441	−0.247	−0.435 *

* *p* < 0.05 was considered statistically significant. FMD, flow-mediated dilatation; CAED, carotid artery end diastolic diameter; left CIMT, left carotid artery intima-media thickness; right CIMT, right carotid artery intima-media thickness; right baPWV, brachial-ankle pulse wave velocity; left baPWV, left brachial-ankle pulse wave velocity.

**Table 5:** Analysis of covariance of the FMD, WSS, Cluster 1, Cluster 2, and Cluster 3, using age as a covariate.

	Nonplayers	Tennis Players
FMD	8.69 ± 0.52	10.55 ± 0.58 * $R{²}_{ad}$ = 0.367
WSS	7.14 ± 0.49	9.25 ± 0.54 ** $R{²}_{ad}$ = 0.284
C1	−1.04 ± 0.86	1.28 ± 0.96 $R{²}_{ad}$ = 0.038
C2	−1.51 ± 1.01	1.87 ± 1.12 * $R{²}_{ad}$ = 0.341
C3	−1.81 ± 0.52	2.24 ± 0.58 *** $R{²}_{ad}$ = 0.45

The data are presented as mean ± SE. * *p* < 0.05, ** *p* < 0.01, and *** *p* < 0.001 were considered statistically significant. Cluster 1 (C1) represents the cardiac function and is the summary of the z-scores for the HR, SVI, CI, SV, CO, and EF; Cluster 2 (C2) represents the arterial function and is the summary of the z-scores for the FMD, WSS, CIMT, baPWV, ABI, PSV, and CAED; Cluster 3 (C3) represents the body composition function and is the summary of the z-scores for the BMI, BMP, BFM, and LBM.

**Table 6:** Correlation between age and Cluster 1, Cluster 2, and Cluster 3.

	Nonplayers	Tennis Players
Cluster 1	0.162	0.202
Cluster 2	−0.652 **	−0.287
Cluster 3	−0.454 *	0.199

* *p* < 0.05, ** *p* < 0.01 were considered statistically significant. Cluster 1 (C1) represents the cardiac function and is the summary of the z-scores for the HR, SVI, CI, SV, CO, and EF; Cluster 2 (C2) represents the arterial function and is the summary of the z-scores for the FMD, WSS, CIMT, baPWV, ABI, PSV, and CAED; Cluster 3 (C3) represents the body composition function and is the summary of the z-scores for the BMI, BMP, BFM, and LBM.

**Table 7:** Correlation between the FMD and Cluster 1, Cluster 2, and Cluster 3.

	Simple Correlation Analysis		Partial Correlation Analysis Adjusted by Age	
	Nonplayers	Tennis players	Nonplayers	Tennis players
C1	−0.287	−0.227	−0.239	−0.167
C2	0.528 *	0.741 **	0.258	0.714 *
C3	0.247	0.226	−0.011	0.330

* *p* < 0.05, ** *p* < 0.01 were considered statistically significant. Cluster 1 (C1) represents the cardiac function and is the summary of the z-scores for the HR, SVI, CI, SV, CO, and EF; Cluster 2 (C2) represents the arterial function and is the summary of the z-scores for the FMD, WSS, CIMT, baPWV, ABI, PSV, and CAED; Cluster 3 (C3) represents the body composition function and is the summary of the z-scores for the BMI, BMP, BFM, and LBM.

## Data Availability

The data presented in this study are available in the supplementary material.

## Supplementary Materials

The following are available online at https://www.mdpi.com/article/10.3390/ijerph192315749/s1.

## References

[1] World Health Statistics 2021. https://apps.who.int/iris/bitstream/handle/10665/342703/9789240027053-eng.pdf.

[2] Lavie C.J., Ozemek C., Carbone S., Katzmarzyk P.T., Blair S.N. (2019). Sedentary Behavior, Exercise, and Cardiovascular Health. Circ. Res..

[3] Elagizi A., Kachur S., Carbone S., Lavie C.J., Blair S.N. (2020). A Review of Obesity, Physical Activity, and Cardiovascular Disease. Curr. Obes. Rep..

[4] Bull F.C., Al-Ansari S.S., Biddle S., Borodulin K., Buman M.P., Cardon G., Carty C., Chaput J.P., Chastin S., Chou R. (2020). World Health Organization 2020 guidelines on physical activity and sedentary behaviour. Br. J. Sports Med..

[5] Lee D.C., Sui X., Ortega F.B., Kim Y.S., Church T.S., Winett R.A., Ekelund U., Katzmarzyk P.T., Blair S.N. (2010). Comparisons of leisure-time physical activity and cardiorespiratory fitness as predictors of all-cause mortality in men and women. Br. J. Sports Med..

[6] Chen X., Zhang M., Wang L., Huang Z., Zhang W., Wu J. (2022). Association between physical activity energy expenditure and cardiometabolic risk factor clustering among Chinese adults in 2015. Sports Med. Health Sci..

[7] Hallal P.C., Andersen L.B., Bull F.C., Guthold R., Haskell W., Ekelund U. (2012). Global physical activity levels: Surveillance progress, pitfalls, and prospects. Lancet.

[8] Panter J., Guell C., Prins R., Ogilvie D. (2017). Physical activity and the environment: Conceptual review and framework for intervention research. Int. J. Behav. Nutr. Phys. Act..

[9] Garber C.E., Blissmer B., Deschenes M.R., Franklin B.A., Lamonte M.J., Lee I.M., Nieman D.C., Swain D.P. (2011). American College of Sports Medicine position stand. Quantity and quality of exercise for developing and maintaining cardiorespiratory, musculoskeletal, and neuromotor fitness in apparently healthy adults: Guidance for prescribing exercise. Med. Sci. Sports Exerc..

[10] Harden S.M., McEwan D., Sylvester B.D., Kaulius M., Ruissen G., Burke S.M., Estabrooks P.A., Beauchamp M.R. (2015). Understanding for whom, under what conditions, and how group-based physical activity interventions are successful: A realist review. BMC Public Health.

[11] Chomistek A.K., Cook N.R., Flint A.J., Rimm E.B. (2012). Vigorous-intensity leisure-time physical activity and risk of major chronic disease in men. Med. Sci. Sports Exerc..

[12] Pluim B.M., Groppel J.L., Miley D., Crespo M., Turner M.S. (2018). Health benefits of tennis. Br. J. Sports Med..

[13] König D., Huonker M., Schmid A., Halle M., Berg A., Keul J. (2001). Cardiovascular, metabolic, and hormonal parameters in professional tennis players. Med. Sci. Sports Exerc..

[14] Kovacs M.S. (2007). Tennis physiology: Training the competitive athlete. Sports Med..

[15] Pluim B.M., Staal J.B., Marks B.L., Miller S., Miley D. (2007). Health benefits of tennis. Br. J. Sports Med..

[16] Carpes L., Jacobsen A., Domingues L., Jung N., Ferrari R. (2021). Recreational beach tennis reduces 24-h blood pressure in adults with hypertension: A randomized crossover trial. Eur. J. Appl. Physiol..

[17] Libby P. (2021). The changing landscape of atherosclerosis. Nature.

[18] Ross R. (1993). The pathogenesis of atherosclerosis: A perspective for the 1990s. Nature.

[19] Celermajer D.S., Sorensen K.E., Bull C., Robinson J., Deanfield J.E. (1994). Endothelium-dependent dilation in the systemic arteries of asymptomatic subjects relates to coronary risk factors and their interaction. J. Am. Coll. Cardiol..

[20] Anderson L., Oldridge N., Thompson D.R., Zwisler A.D., Rees K., Martin N., Taylor R.S. (2016). Exercise-Based Cardiac Rehabilitation for Coronary Heart Disease: Cochrane Systematic Review and Meta-Analysis. J. Am. Coll. Cardiol..

[21] Green D.J., Smith K.J. (2017). Effects of Exercise on Vascular Function, Structure, and Health in Humans. Cold Spring Harb. Perspect. Med..

[22] Ashor A.W., Lara J., Siervo M., Celis-Morales C., Oggioni C., Jakovljevic D.G., Mathers J.C. (2014). Exercise modalities and endothelial function: A systematic review and dose-response meta-analysis of randomized controlled trials. Sports Med..

[23] Green D.J., Hopman M.T., Padilla J., Laughlin M.H., Thijssen D.H. (2017). Vascular Adaptation to Exercise in Humans: Role of Hemodynamic Stimuli. Physiol. Rev..

[24] Walker A.E., Kaplon R.E., Pierce G.L., Nowlan M.J., Seals D.R. (2014). Prevention of age-related endothelial dysfunction by habitual aerobic exercise in healthy humans: Possible role of nuclear factor κB. Clin. Sci..

[25] Knowlton A.A., Lee A.R. (2012). Estrogen and the cardiovascular system. Pharmacol. Ther..

[26] Nappi R.E., Chedraui P., Lambrinoudaki I., Simoncini T. (2022). Menopause: A cardiometabolic transition. Lancet Diabetes Endocrinol..

[27] Faul F., Erdfelder E., Lang A.G., Buchner A. (2007). G*Power 3: A flexible statistical power analysis program for the social, behavioral, and biomedical sciences. Behav. Res. Methods.

[28] Agrotou S., Karatzi K., Papamichael C., Fatouros I., Mitrakou A., Zakopoulos N., Dimopoulos A., Stamatelopoulos K. (2013). Effects of chronic anaerobic training on markers of sub-clinical atherosclerosis. Hellenic. J. Cardiol..

[29] Yu C.-Y., Lo Y.-H., Chiou W.-K. (2003). The 3D scanner for measuring body surface area: A simplified calculation in the Chinese adult. Appl. Ergon..

[30] Malek A.M., Alper S.L., Izumo S. (1999). Hemodynamic shear stress and its role in atherosclerosis. JAMA.

[31] Corretti M.C., Anderson T.J., Benjamin E.J., Celermajer D., Charbonneau F., Creager M.A., Deanfield J., Drexler H., Gerhard-Herman M., Herrington D. (2002). Guidelines for the ultrasound assessment of endothelial-dependent flow-mediated vasodilation of the brachial artery: A report of the International Brachial Artery Reactivity Task Force. J. Am. Coll. Cardiol..

[32] Jackson M.J., Roche D.M., Amirabdollahian F., Koehn S., Khaiyat O.A. (2019). The Musculoskeletal Health Benefits of Tennis. Sports health.

[33] Andersen L.B., Harro M., Sardinha L.B., Froberg K., Ekelund U., Brage S., Anderssen S.A. (2006). Physical activity and clustered cardiovascular risk in children: A cross-sectional study (The European Youth Heart Study). Lancet.

[34] Oja P., Kelly P., Pedisic Z., Titze S., Bauman A., Foster C., Hamer M., Hillsdon M., Stamatakis E. (2017). Associations of specific types of sports and exercise with all-cause and cardiovascular-disease mortality: A cohort study of 80,306 British adults. Br. J. Sports Med..

[35] Paffenbarger R.S., Hyde R.T., Wing A.L., Lee I.M., Jung D.L., Kampert J.B. (1993). The association of changes in physical-activity level and other lifestyle characteristics with mortality among men. N. Engl. J. Med..

[36] Cahill P.A., Redmond E.M. (2016). Vascular endothelium – Gatekeeper of vessel health. Atherosclerosis.

[37] Celermajer D.S., Sorensen K.E., Gooch V.M., Spiegelhalter D.J., Miller O.I., Sullivan I.D., Lloyd J.K., Deanfield J.E. (1992). Non-invasive detection of endothelial dysfunction in children and adults at risk of atherosclerosis. Lancet.

[38] Yeboah J., Folsom A.R., Burke G.L., Johnson C., Polak J.F., Post W., Lima J.A., Crouse J.R., Herrington D.M. (2009). Predictive value of brachial flow-mediated dilation for incident cardiovascular events in a population-based study: The multi-ethnic study of atherosclerosis. Circulation.

[39] Kitta Y., Obata J.E., Nakamura T., Hirano M., Kodama Y., Fujioka D., Saito Y., Kawabata K., Sano K., Kobayashi T. (2009). Persistent impairment of endothelial vasomotor function has a negative impact on outcome in patients with coronary artery disease. J. Am. Coll. Cardiol..

[40] Shechter M., Shechter A., Koren-Morag N., Feinberg M.S., Hiersch L. (2014). Usefulness of brachial artery flow-mediated dilation to predict long-term cardiovascular events in subjects without heart disease. Am. J. Cardiol..

[41] Thijssen D.H., Carter S.E., Green D.J. (2015). Arterial structure and function in vascular ageing: Are you as old as your arteries?. J. Physiol..

[42] Calabresi L., Gomaraschi M., Simonelli S., Bernini F., Franceschini G. (2015). HDL and atherosclerosis: Insights from inherited HDL disorders. Biochim. Biophys. Acta.

[43] Casanova G., Bossardi Ramos R., Ziegelmann P., Spritzer P.M. (2015). Effects of low-dose versus placebo or conventional-dose postmenopausal hormone therapy on variables related to cardiovascular risk: A systematic review and meta-analyses of randomized clinical trials. J. Clin. Endocrinol. Metab..

[44] Cunnane C.V., Cunnane E.M., Walsh M.T. (2017). A Review of the Hemodynamic Factors Believed to Contribute to Vascular Access Dysfunction. Cardiovasc. Eng. Technol..

[45] Andersson M., Lantz J., Ebbers T., Karlsson M. (2017). Multidirectional WSS disturbances in stenotic turbulent flows: A pre- and post-intervention study in an aortic coarctation. J. Biomech..

[46] Randles A., Frakes D.H., Leopold J.A. (2017). Computational Fluid Dynamics and Additive Manufacturing to Diagnose and Treat Cardiovascular Disease. Trends Biotechnol.

[47] Zhao J., Yan H.M., Li Y., Wang J., Han L., Wang Z.H., Tang M.X., Zhang W., Zhang Y., Zhong M. (2015). Pitavastatin calcium improves endothelial function and delays the progress of atherosclerosis in patients with hypercholesterolemia. J. Zhejiang Univ. Sci. B.

[48] LV Y., Xiong K., Zhao L. (2016). Effects of ACE D/I Polymorphism and Resistance Training on Vascular Endothelial Function in Postmenopausal Women. J. Beijing Sport Univ..

[49] Tanahashi K., Kosaki K., Sawano Y., Yoshikawa T., Tagawa K., Kumagai H., Akazawa N., Maeda S. (2017). Impact of Age and Aerobic Exercise Training on Conduit Artery Wall Thickness: Role of the Shear Pattern. J. Vasc. Res..

[50] Shuto E., Taketani Y., Tanaka R., Harada N., Isshiki M., Sato M., Nashiki K., Amo K., Yamamoto H., Higashi Y. (2009). Dietary phosphorus acutely impairs endothelial function. J. Am. Soc. Nephrol..

[51] Groppel J., DiNubile N. (2009). Tennis: For the health of it!. Physician Sportsmed..

[52] Sundstrup E., Jakobsen M.D., Brandt M., Jay K., Aagaard P., Andersen L.L. (2016). Strength Training Improves Fatigue Resistance and Self-Rated Health in Workers with Chronic Pain: A Randomized Controlled Trial. BioMed Res. Int..

[53] Price K.J., Gordon B.A., Bird S.R., Benson A.C. (2016). A review of guidelines for cardiac rehabilitation exercise programmes: Is there an international consensus?. Eur. J. Prev. Cardiol..

[54] Schroeder E.C., Franke W.D., Sharp R.L., Lee D.C. (2019). Comparative effectiveness of aerobic, resistance, and combined training on cardiovascular disease risk factors: A randomized controlled trial. PLoS ONE.

[55] Kovacs M.S. (2006). Applied physiology of tennis performance. Br. J. Sports Med..

[56] Fernandez J., Mendez-Villanueva A., Pluim B.M. (2006). Intensity of tennis match play. Br. J. Sports Med..

[57] Reid M., Duffield R. (2014). The development of fatigue during match-play tennis. Br. J. Sports Med..

[58] Torres-Luque G., SÁNchez-Pay A., Belmonte J.B., RamóN M.M. (2011). Functional aspects of competitive tennis. J. Hum. Sport Exerc..

[59] Houston T.K., Meoni L.A., Ford D.E., Brancati F.L., Cooper L.A., Levine D.M., Liang K.Y., Klag M.J. (2002). Sports ability in young men and the incidence of cardiovascular disease. Am. J. Med..

[60] Stamatakis E., Kelly P., Titze S., Pedisic Z., Bauman A., Foster C., Hamer M., Hillsdon M., Oja P. (2017). The associations between participation in certain sports and lower mortality are not explained by affluence and other socioeconomic factors. Br. J. Sports Med..

